# The critical role of pancreatic stone protein/regenerating protein in sepsis-related multiorgan failure

**DOI:** 10.3389/fmed.2023.1172529

**Published:** 2023-05-05

**Authors:** Ping Hu, Yuan hua Lu, Wei Deng, Qi Li, Ning Zhao, Qiang Shao, Ling Wu, Xu zhen Wang, Ke jian Qian, Fen Liu

**Affiliations:** ^1^Department of Critical Care Medicine, The First Affiliated Hospital of Nanchang University, Nanchang, Jiangxi, China; ^2^Medical Innovation Center, The First Affiliated Hospital of Nanchang University, Nanchang, Jiangxi, China

**Keywords:** sepsis, neutrophils, infiltration, pancreatic stone protein, multiple organ dysfunction syndrome

## Abstract

**Introduction:**

Multiple organ dysfunction syndrome (MODS) is common in patients with sepsistic admitted to an intensive care unit (ICU) and greatly increases mortality. Pancreatic stone protein/regenerating protein (PSP/Reg) is a type of C-type lectin protein that is overexpressed during sepsis. This study aimed to evaluate the potential involvement of PSP/Reg in MODS development in patients with sepsis.

**Materials and methods:**

The relationship between circulating PSP/Reg levels, patient prognosis, and progression to MODS was analyzed in patients with sepsis admitted to the ICU of a general tertiary hospital. Furthermore, to examine the potential involvement of PSP/Reg in sepsis-induced MODS, a septic mouse model was established per the cecal ligation and puncture procedure, randomized into three groups, and subjected to a caudal vein injection of recombinant PSP/Reg at two different doses and phosphate-buffered saline. Survival analyses and disease severity scoring were performed to evaluate the survival status of the mice; enzyme-linked immunosorbent assays were performed to detect the levels of inflammatory factors and organ-damage markers in murine peripheral blood; terminal deoxynucleotidyl transferase dUTP nick end labeling (TUNEL) staining was performed to measure apoptosis levels in lung, heart, liver, and kidney tissue sections and to visualize the degree of organ damage in the mouse model; myeloperoxidase activity assay, immunofluorescence staining, and flow cytometry were performed to detect neutrophil infiltration levels in vital murine organs and the activation indexes of neutrophils.

**Results and discussion:**

Our findings indicated that Circulating PSP/Reg levels were correlated with patient prognosis and sequential organ failure assessment scores. Furthermore, PSP/Reg administration increased disease severity scores, shortened survival time, increased the TUNEL-positive staining rate, and increased the levels of inflammatory factors, organ-damage markers, and neutrophil infiltration in the organs. Neutrophils can be activated by PSP/Reg to an inflammatory state, both *in vivo* and *in vitro*, which is characterized by the increased levels of intercellular adhesion molecule 1 and CD29.

**Conclusion:**

Patient prognosis and progression to MODS can be visualized by monitoring PSP/Reg levels upon ICU admission. Additionally, PSP/Reg administration in animal models exacerbates the inflammatory response and severity of multiorgan damage, which may be accomplished by promoting the inflammatory state of neutrophils.

## 1. Introduction

Sepsis was initially considered a systemic inflammatory response syndrome mediated by the release of massive amounts of inflammatory mediators; however, subsequent clinical trials targeting inflammatory suppression failed to reduce the mortality associated with sepsis (1–3). This failure has prompted a re-evaluation of the underlying mechanisms of sepsis and led to a growing appreciation of the complex interplay between host defense mechanisms, immune activation, and tissue injury that contributes to the development of sepsis and multiple organ dysfunction syndrome (MODS). The Third International Consensus Definitions for Sepsis and Septic Shock (Sepsis-3), published in 2016, defines sepsis as a life-threatening organ dysfunction induced by a dysregulated host response to infections, reaffirming the significance of the development of organ dysfunction (4) because the severity of organ dysfunction and the number of failed organs are closely correlated with the survival probability of patients. It has been estimated that 15% of patients admitted to an intensive care unit (ICU) experience MODS, which, depending on the number of dysfunctional organs, is associated with a mortality rate as high as 30–100% (5). However, the mechanism by which of sepsis triggers organ dysfunction is not fully understood, making MODS the leading cause of morbidity and mortality in patients admitted to the ICU (5).

## 2. Materials and methods

### 2.1. Study design and patients

In the current prospective study, adult patients with sepsis admitted to the ICU of the First Affiliated Hospital of Nanchang University from September 2021 to October 2022 were included, and some patients with potential bias were excluded (Supplementary Figure S1). Clinical data and blood samples were collected upon admission. The blood samples were centrifuged at 4°C (1,000 × *g*, 15 min) within 30 min of the collection to get the serum, which was then stored at −80°C for subsequent analysis. The study endpoints were mortality 28 days after admission and progression to MODS 48–72 h after admission. MODS was identified when the Sequential Organ Failure Assessment (SOFA) score was ≥5 points.

This study was approved by the Ethics Committee of the First Affiliated Hospital of Nanchang University (approval number: NC-2022-1-004), and each patient signed an informed consent form prior to participation.

### 2.2. Mice and septic model

C57BL/6 male mice aged 8–10 weeks and weighing 20–24 g were purchased from Vital River Lab Rotary (Zhejiang, China) and housed under specific pathogen-free conditions. All the experiments were performed in accordance with the regulations of the Experimental Animal Welfare and Ethics Committee of the First Affiliated Hospital of Nanchang University. Prior to the experiment, the mice were acclimated to a controlled indoor environment for over 1 week without food or water restrictions, followed by cecal ligation and puncture (CLP) for sepsis induction (6). The specific steps are briefly described below. After pentobarbital anesthesia, a midline incision of 1–2 cm was made under sterile conditions to expose the cecum, followed by the ligation of the cecal ileocecal valve using 6–0 nonabsorbable sutures between the third and fourth caudal vessels of the cecum. Subsequently, the cecum was punctured with an 18G needle to gently squeeze a small amount of intestinal content into the murine abdominal cavity, which was then closed with sutures after the intestine was incorporated. Postoperatively, the mice were placed on a heating pad, injected with 1 mL saline, and observed until the emergence of anesthesia.

Twenty-four hours after CLP, the health status of the mice was evaluated in a blinded manner based on the murine sepsis score (7). The scoring system comprises seven elements: appearance, level of consciousness, activity, response to stimuli, eyes, respiration rate, and respiration quality.

### 2.3. Animal treatments

Following CLP, the mice were randomized into phosphate-buffered saline (PBS) (control), PSP-40, and PSP-400 groups (*n* = 5 per group). Recombinant pancreatic stone protein/regenerating protein (PSP/Reg) (Sino Biological) was dissolved in PBS of 100 μL, and the solution was injected into the caudal vein 30 min after CLP at 40 and 400 ng/kg. Mice injected with low-dose PSP/Reg corresponded to patients with systemic infections, whereas high-dose PSP/Reg was the median amount in patients with sepsis (8). Control mice were administered PBS of the same volume, and all mice were euthanized 24 h post-treatment, and plasma, lung, liver, heart, and kidney samples were collected. A portion of the samples was utilized for immediate tissue myeloperoxidase (MPO) activity testing, whereas the remaining portion was promptly frozen and stored at −80°C for subsequent analysis.

### 2.4. Survival study

To evaluate the effect of PSP/Reg in a septic mouse model, 26 mice were randomized into three groups 30 min after CLP and injected with PSP/Reg (40 ng/kg) (*n* = 10), PSP/Reg (400 ng/kg) (*n* = 10), or an equivalent volume of PBS (*n* = 6) in the caudal vein. Over the subsequent 7 days, the survival rate was assessed every 2 h.

### 2.5. Measurement of PSP/Reg, cytokines, and organ-damage markers

The concentration of PSP/Reg in the serum samples was determined using a commercially available Human REG1α ELISA Kit (Wuhan Fine Biotech) according to the manufacturer’s protocol. The laboratory technicians who performed the assays were blinded to the clinical information. The levels of cytokines (tumor necrosis factor-α, interleukin [IL]-6, and IL-1β) and organ-damage markers (lactate dehydrogenase, creatinine, and troponin I) were quantified using ELISA assay kits from R&D Systems and BD Life Science, respectively, following the manufacturer’s instructions.

### 2.6. Immunofluorescence staining and terminal deoxynucleotidyl transferase dUTP nick end labeling (TUNEL)

Tissue samples were fixed in 4% paraformaldehyde, embedded into the optimal-cutting-temperature compound, and cut into 5-μm sections. The sections were then incubated in a blocking solution (PBS containing 10% goat serum and 1% bovine serum albumin) for 30 min, followed by overnight co-incubation at 4°C with the anti-LY6G antibody (Abcam). After washing, the sections were incubated with the fluorescein isothiocyanate-conjugated secondary antibody (Proteintech) for 2 h, and the cell nuclei were labeled with 4′,6-diamidino-2-phenylindole (Bioss).

The TUNEL assay was performed using One-Step TUNEL Apoptosis Assay Kit (Beyotime Biotechnology) following the manufacturer’s instructions. Tissue sections were fixed in 4% paraformaldehyde, permeabilized with 0.1% Triton X-100 for 10 min, and incubated with the TUNEL reaction mixture for 1 h at 37°C in the dark. Cell nuclei were visualized with 4′,6-diamidino-2-phenylindole. Images were captured using an Olympus IX71 microscope and quantified using the ImageJ software.

### 2.7. MPO activity assay

MPO activity in murine lungs, hearts, livers, and kidneys was evaluated by performing a tissue MPO activity assay (Elabscience Biotechnology Co. Ltd.) according to the manufacturer’s instructions. MPO activity was expressed as the optical density at 460 nm per mg of protein.

### 2.8. Determination of wet/dry ratio in lung tissue

Fresh lung tissue was placed on sterile gauze and weighed to obtain the wet weight. The tissue was then placed in an oven at 60°C for 72 h to obtain the dry weight, which was measured three times. The wet/dry ratio was calculated as the ratio of the wet weight to the dry weight.

### 2.9. Neutrophil isolation and culture

A mouse bone marrow neutrophil isolation kit (Solarbio) was used to isolate neutrophils from the mouse bone marrow. The specific steps are as follows. The mouse thigh bone was first rinsed with 75% ethanol and PBS and then with the 10% fetal bovine serum-supplemented Roswell Park Memorial Institute 1,640 medium after both ends of the thigh bone were sheared to obtain bone cells. Next, density gradient centrifugation was performed according to the manufacturer’s instructions after erythrocyte lysis to obtain neutrophils, the morphology of which was observed by performing Wright-Giemsa staining. Flow cytometry (FCM) analysis revealed that the purity of the neutrophils was >90%. Subsequently, the isolated neutrophils were cultured in the complete Roswell Park Memorial Institute medium (10% PBS, 1% penicillin/streptomycin, 1% glutamine), and equivalent amounts of 40 ng/mL or 400 ng/ml PSP/Reg or PBS for 24 h of stimulation. The next day the Neutrophils were collected for further analysis.

### 2.10. FCM

Phycoerythrin-conjugated anti-intercellular adhesion molecule 1 (ICAM-1) (BD Biosciences) and fluorescein isothiocyanate-conjugated anti-CD29 antibodies (BD Biosciences) were used to detect the levels of surface antigens on neutrophils. After co-incubation at room temperature for 30 min, the cells were washed thrice with PBS containing 2% bovine serum albumin. FCM analysis was performed using an LSRFortessa cell analyzer (BD Biosciences), and the samples were analyzed using the FlowJo software.

### 2.11. Statistical analysis

Continuous variables in the clinical data were non-parametrically distributed and are, therefore, represented as the median (interquartile range [IQR]) or number (percentage). The Mann–Whitney U test was performed to compare differences in the median values of continuous clinical variables between the two groups, and the Kruskal–Wallis test was performed to compare PSP/Reg levels among the groups according to the SOFA score. The Spearman rank correlation test was performed to analyze the correlation between circulating PSP/Reg levels and organ support therapy using human data. Logistic regression and receiver operating characteristic (ROC) analyses were performed to assess whether circulating PSP/Reg was a predictive factor for MODS development (48–72 h after ICU admission) and 28-day mortality. Continuous variables in the *in vitro* and *in vivo* experiments were normally distributed and are described as the mean ± standard error of the mean. Statistical significance was assessed using Student’s *t*-test or one- or two-way analysis of variance, as appropriate, to identify any differences within the groups. Kaplan–Meier survival curves were used to compare survival times, and the log-rank test was performed to compare differences between the groups.

## 3. Results

### 3.1. Increased circulating PSP/Reg levels in patients with sepsis admitted to the ICU were associated with MODS

To determine the clinical relevance of circulating PSP/Reg levels, 141 adult patients with sepsis who were admitted to the ICU were prospectively recruited. The patients’ clinical characteristics are presented in Table 1. Among the included patients, 102 (72%) survived (survival group) and 39 (28%) died (death group), with males outnumbering females in each subgroup. A total of 72 cases (51%) presented with community-acquired infections, whereas 69 (49%) with hospital-acquired infections. Most patients (55%) had Gram-negative infections, and the most common site of infection was the lungs (63%). The average length of ICU stay was 11 days (9–14 days).

**Table 1:** Baseline characteristics of patients included in the study.

Characteristic	Total (*n* = 141)	Survivors (*n* = 102)	Non-survivors (*n* = 39)	*p*-values
Age (years)	61 (50–72)	60 (49–71)	63 (51–74)	0.80
Male, *n* (%)	91 (64)	69 (68)	22 (57)	0.21
ICU stay (days)	11 (9–14)	9 (7–11)	12 (10–14)	0.001
Site of infection, *n* (%)				
Abdomen	35 (25)	25 (25)	10 (26)	0.88
Respiratory tract	89 (63)	67 (66)	22 (56)	0.307
Urinary tract	2 (2)	2 (2)	None	–
Skin/Soft tissue	5 (4)	3 (3)	2 (5)	0.53
Others	10 (7)	5 (5)	5 (13)	0.1
Community/Hospital infections, *n* (%)	72 (51)	65 (64)	7 (18)	<0.001
Pathogen, *n* (%)				
Gram-positive	50 (35)	39 (38)	11 (28)	0.26
Gram-negative	78 (55)	56 (55)	22 (56)	0.872
Both	7 (5)	3 (3)	4 (10)	0.07
Fungi	6 (4)	4 (4)	2 (1)	0.75
Score (IQR)				
APACHE II	23 (16–33)	21 (16–31)	24 (19–36)	<0.001
SOFA (day 1)	6 (3–10)	4 (3–8)	7 (4–10)	<0.001
Organ failure, *n* (%)	87 (62)	57 (56)	30 (77)	0.028
Renal replacement therapy, *n* (%)	12 (9)	6 (6)	6 (15)	0.07
Vasopressors, *n* (%)	85 (60)	66 (65)	19 (49)	0.08
Mechanical ventilation, *n* (%)	93 (66)	71 (69)	22 (56)	0.139
AKI within 7 days, *n* (%)	43 (30)	28 (27)	15 (38)	0.204

**Figure 1:**
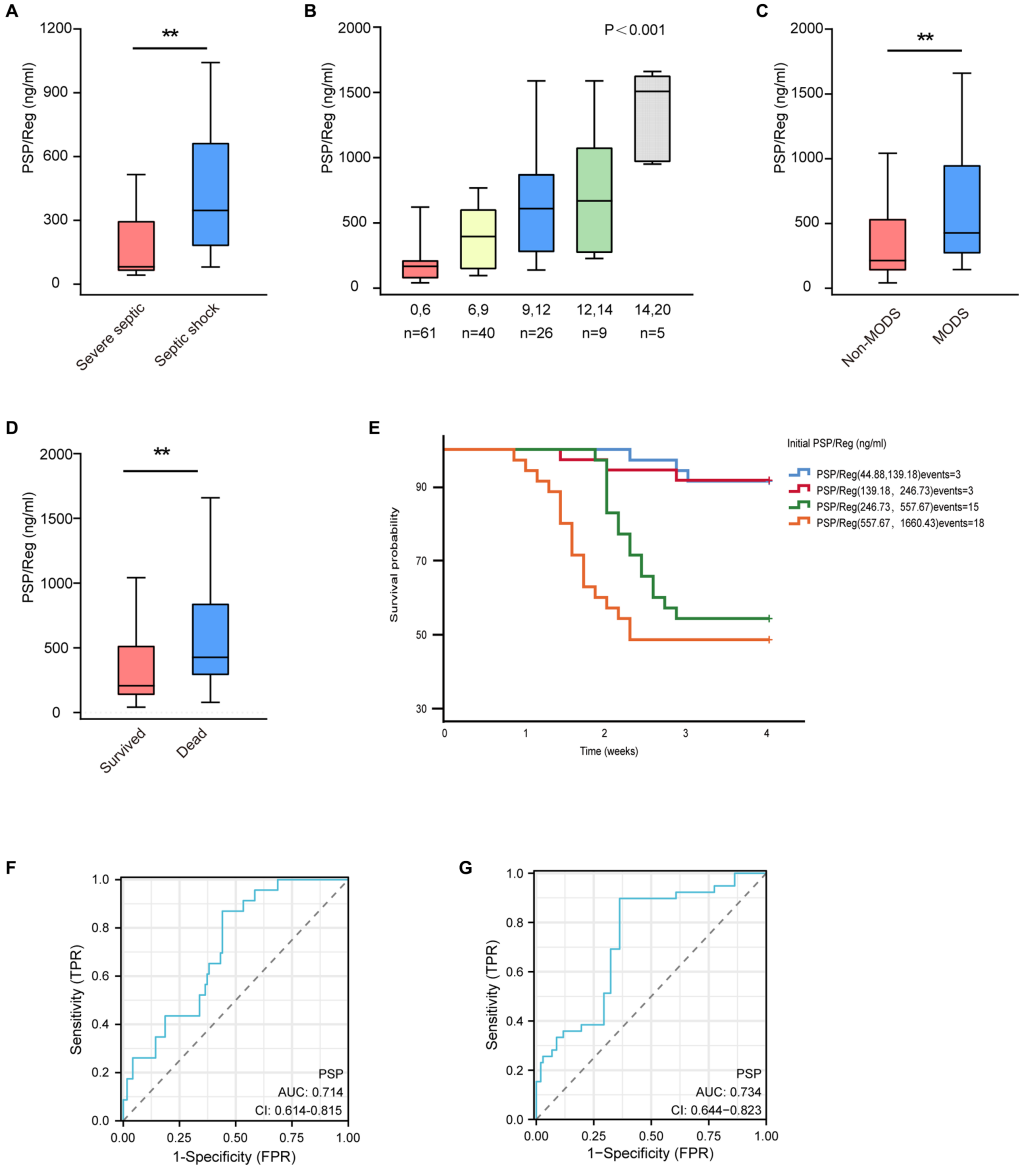
Correlation between circulating Pancreatic stone protein/regenerating protein (PSP/Reg) levels and prognosis as well as progression to multiple organ dysfunction syndrome (MODS) in patients with sepsis admitted to an intensive care unit. **(A)** Circulating PSP/Reg levels were compared according to disease severity in patients. Mann–Whitney *U* test showed a significant increase in PSP/Reg levels in patients with septic shock compared with an increase in those with severe sepsis (*p* < 0.001). **(B)** PSP/Reg levels were evaluated in patients stratified by Sequential Organ Failure Assessment (SOFA) scores, and Kruskal–Wallis test showed a significant increase in PSP/Reg levels (median and interquartile range) with increasing SOFA scores (*p* < 0.001). Mann–Whitney U test indicated significant differences in PSP/Reg levels between patients with and without MODS **(C)** and between survivors and non-survivors of sepsis. **(D)** ^*^p < 0.05, ^**^p < 0.01. **(E)** According to the quartile division of PSP/Reg at admission, the Kaplan–Meier survival curve for 28 days is shown. The receiver operating characteristic curve analysis of PSP/Reg for the prediction of MODS **(F)** and 28d mortality **(G)**.

Overall, the data revealed that the circulating PSP/Reg levels in patients with septic shock improved significantly compared with those in patients with severe sepsis (median, 347.1 ng/mL; [IQR, 179.1–665.2 ng/mL] vs. 82 ng/mL [IQR, 62–297.7 ng/mL]; *p* < 0.001) (Figure 1A). In addition, initial PSP/Reg levels correlated with initial SOFA scores (*p* < 0.001) (Figure 1B).

The Spearman rank correlation test showed a strong correlation between circulating PSP/Reg levels and dependence on organ support therapies among patients with a high demand for vasopressors at admission (*r* = 0.496; *p* < 0.001) and those who needed the long-term administration of vasopressors (*r* = 0.545; *p* < 0.001), mechanical ventilation (*r* = 0.607; *p* < 0.01), or renal replacement therapy (*r* = 0.360; *p* = 0.015).

Patients with MODS (SOFA scores ≥5) exhibited higher circulating PSP/Reg levels compared with patients without MODS (median, 427.2 ng/mL [IQR, 268–951.2 ng/mL] vs. 213.8 ng/mL [IQR, 136.8–536.4 ng/mL]; *p* = 0.001) (Figure 1C). Similarly, patients who died within 28 days of ICU stay exhibited higher circulating PSP/Reg levels than did surviving patients (median, 427.2 ng/mL [IQR, 289–842.1 ng/mL] vs. 208.1 ng/mL [IQR, 135.3–516.2 ng/mL]; *p* < 0.001) (Figure 1D).

Performing the Kaplan–Meier analysis of PSP/Reg values stratified by quartiles revealed that PSP/Reg plasma levels at admission exhibited a strong correlation with the 28-day mortality rate. Furthermore, the survival rate of patients with PSP/Reg levels exceeding the third quartile (246.73 ng/mL) sharply declined during the second week of sepsis (Figure 1E). These findings indicated that the circulating PSP/Reg levels reflected disease severity and that the presence of PSP/Reg might lead to MODS and mortality, especially at high levels.

After adjusting for age and sex, the log regression model showed that the circulating PSP/Reg level was an independent risk factor for progression to MODS 48–72 h after admission and 28-day mortality (odds ratio: 1.012 [1.003, 1.020], *p* = 0.005; odds ratio: 1.006 [1.002, 1.010], *p* < 0.001). The ROC analysis also revealed that it was a predictor of progression to MODS (area under the ROC curve, 0.714; *p* = 0.001) (Figure 1F) and 28-day mortality (area under the ROC curve, 0.734; *p* < 0.001) (Figure 1G).Furthermore, sub-analysis of severe sepsis and septic shock revealed that the predictive value of PSP/Reg level was confirmed (Supplementary Tables S1 and S2).

### 3.2. PSP/Reg aggravated severity scores and shortened survival in septic mice

**Figure 2:**
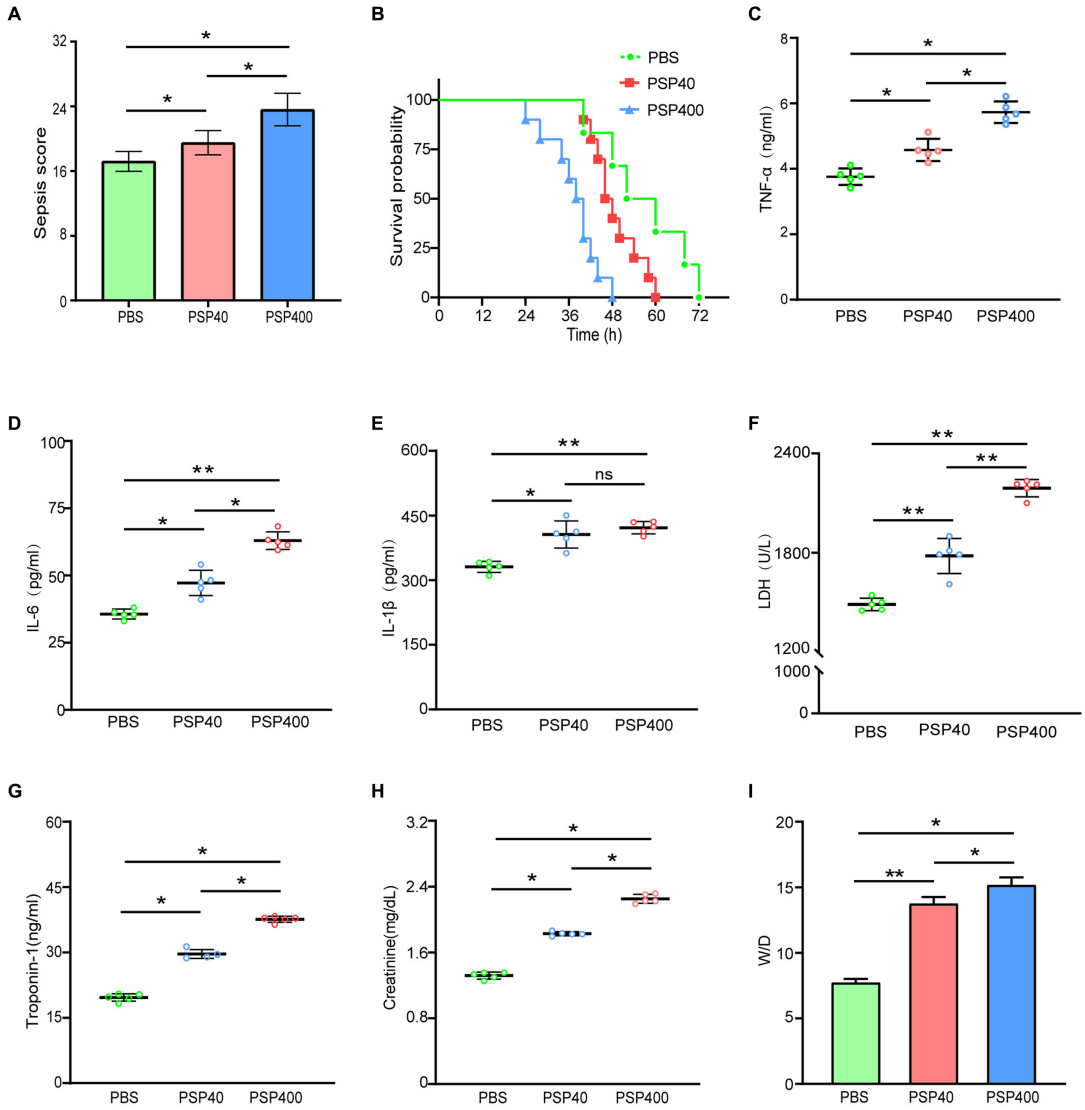
Pancreatic stone protein/regenerating protein (PSP/Reg) exacerbates multiple organ failure in a cecal ligation and puncture (CLP) mouse model. Mice were randomly selected to undergo CLP surgery and were intravenously administered with phosphate-buffered saline (PBS), PSP/Reg (40 ng/kg), or PSP/Reg (400 ng/kg) 30 min later. **(A)** Murine sepsis score at 24 h after CLP was significantly increased by PSP/Reg treatment by one-way analysis of variance (ANOVA). **(B)** Survival curves and log-rank test showed that compared with PBS, PSP/Reg significantly shortened the survival time of the mice. Serum levels of inflammatory cytokines and organ-damage markers were evaluated at 24 h after CLP. **(C)** tumor necrosis factor-α levels (ng/mL), **(D)** interleukin (IL)-6 levels (pg/mL), **(E)** IL-1β levels (pg/mL), **(F)** lactate dehydrogenase (U/L), **(G)** troponin I (ng/mL), **(H)** creatinine (mg/dL), and **(I)** lung wet/dry (W/D) ratio were measured. Data are expressed as the mean ± standard error of the mean and analyzed by one-way ANOVA. ^*^p < 0.01, ^**^p < 0.001, compared with the PBS group.

To investigate the role of PSP/Reg in inflammation in early-stage sepsis, mice were treated with two doses of PSP/Reg 30 min after successful modeling. It was revealed that 24 h after CLP,the disease severity scores of PSP-40 mice were evidently higher compared with those of PBS mice but were visibly lower compared with those of PSP-400 mice (*p* < 0.01), suggesting that PSP/Reg increased the disease severity score of the mice in a dose-depended manner (Figure 2A). Survival analyses were performed to further verify the long-term effect of PSP/Reg on animal models, which showed that the median survival time was significantly shorter in PSP-40 mice compared with that in PBS mice (48 h 95% confidence interval [CI]: 41.86–50.13 vs. 52 h 95% CI: 37.59–66.4, *p* < 0.01), and PSP-400 mice showed the shortest median survival time (48 h 95% CI: 41.86–50.13 vs. 38 h 95% CI: 33.86–42.13, *p* < 0.001) (Figure 2B).

### 3.3. PSP/Reg exacerbated systemic inflammation and multiorgan damage in mouse models

Levels of inflammatory factors reflect the level of systemic inflammation. In this study, compared with PBS mice, PSP-40 mice exhibited significantly increased levels of the inflammatory factors tumor necrosis factor-α, IL-6, and Il-1β. High-dose PSP further increased tumor necrosis factor-α and IL-6 levels but did not further exacerbate IL-1β levels (Figures 2C–E).

Key parameters reflecting multiorgan damage were also measured. It was found that the plasma levels of lactate dehydrogenase (1781 ± 105.4 U/L), troponin I (29.65 ± 1.01 ng/mL), and creatinine (1.82 ± 0.02 mg/dL) in PSP-40 mice were indicative of tissue damage in the liver, heart, and kidney, respectively, compared with those in PBS mice (1,488 ± 37.49 U/L; 19.66 ± 0.85 ng/mL; 1.31 ± 0.04 mg/dL, respectively), and that organ damage was further exacerbated among PSP-400 mice (Figures 2F–H). Additionally, PSP/Reg administration (even at a low dose) resulted in a two-fold increase in the lung wet/dry ratio compared with PBS treatment (Figure 2I). These findings suggested that PSP/Reg aggravated organ damage in a dose-dependent manner in mouse models.

**Figure 3:**
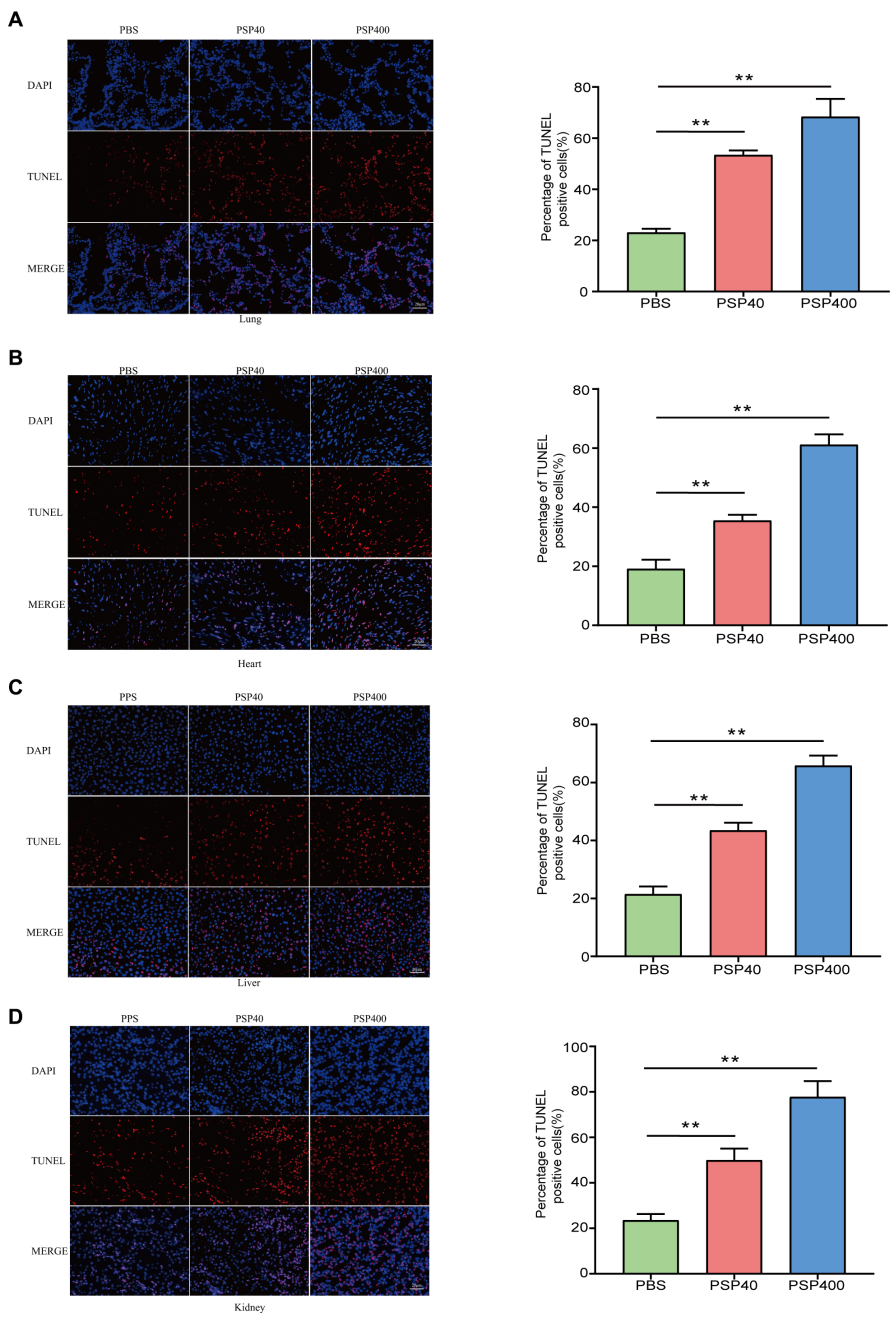
Pancreatic stone protein/regenerating protein exacerbated the level of cellular apoptosis in multiple organ tissues of cecal ligation and puncture (CLP) mice. After 24 h of CLP surgery, terminal deoxynucleotidyl transferase dUTP nick end labeling (TUNEL) staining was performed to assess the level of cellular apoptosis (represented by red fluorescence) in lung **(A)**, heart **(B)**, liver **(C)**, and kidney **(D)** tissue sections and quantified using the ImageJ software with the TUNEL-positive percentage analyzed by one-way analysis of variance. ^*^p < 0.01, ^**^p < 0.001 compared with the phosphate-buffered saline group.

An independent analysis measuring tissue damage was performed on the TUNEL-stained lung, heart, liver, and kidney tissue sections. The lung, heart, liver, and kidney tissues collected from mice injected with PSP/Reg showed a higher TUNEL-positive staining rate than those collected from PBS-treated mice (Figures 3A–D).

### 3.4. PSP/Reg exacerbated neutrophil tissue infiltration and activation *in vivo*

**Figure 4:**
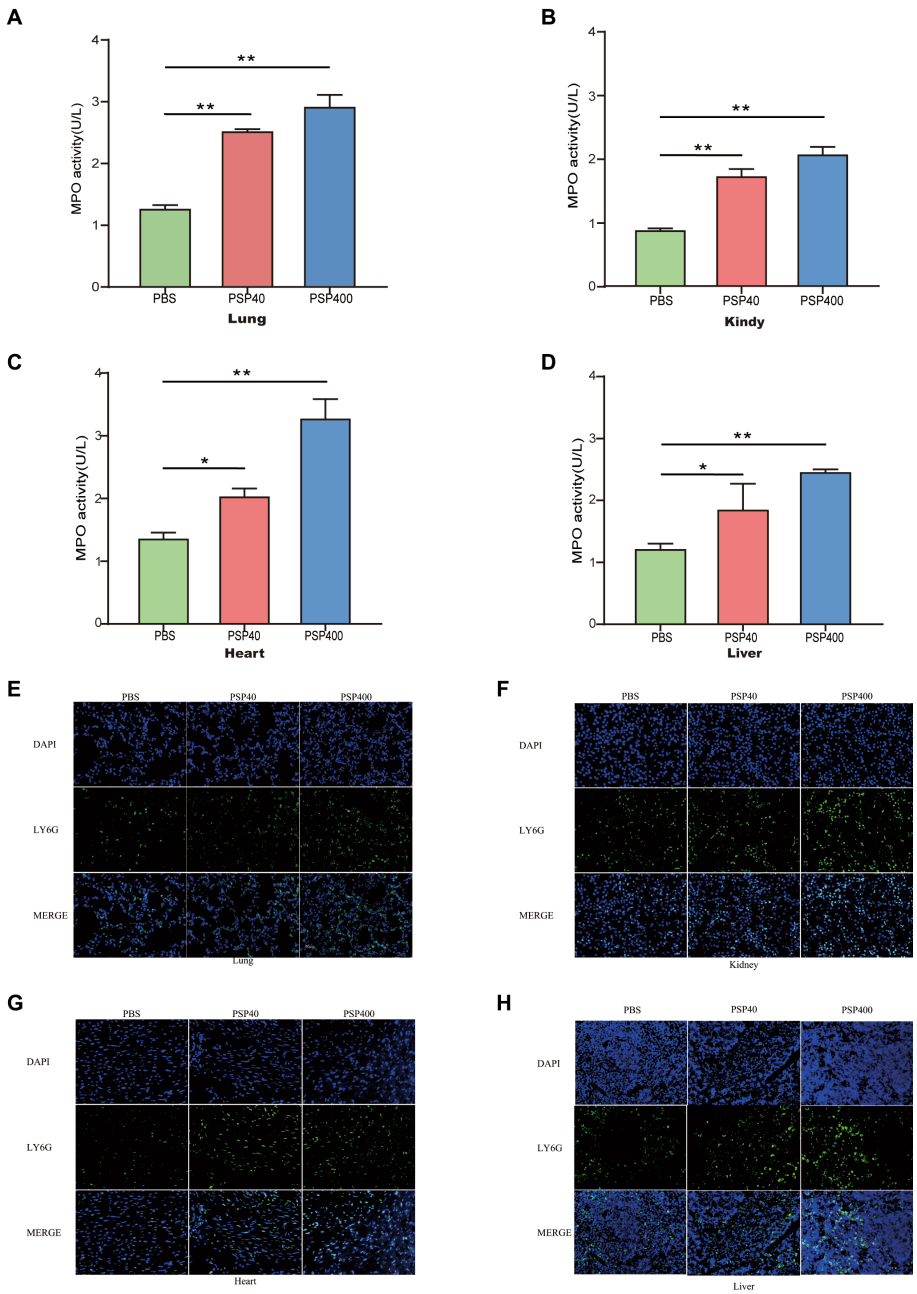
Pancreatic stone protein/regenerating protein (PSP/Reg) increased neutrophil infiltration in multiple organs of cecal ligation and puncture (CLP) mice. The level of myeloperoxidase (MPO) activity and neutrophil (Ly6G+) infiltration in multiple organ tissues were measured 24 h after CLP in mice treated with PSP/Reg. **(A)**: lung tissue MPO activity, **(B)**: kidney tissue MPO activity, **(C)**: heart tissue MPO activity, and **(D)**: liver tissue MPO activity. The data were analyzed by one-way analysis of variance. ^*^p < 0.01, ^**^p < 0.001 compared with the PBS group. Representative images of Ly6G+ (green fluorescence) areas in lung **(E)**, kidney **(F)**, heart **(G)**, and liver **(H)** tissues. Scale bar, 20 mm.

Given the aggravating effect of PSP/Reg on multiorgan damage in the CLP model, the potential mechanism underlying the exacerbation was further explored. Neutrophil infiltration in important organs is a crucial cause of multiple-organ dysfunction and is associated with the severity of organ damage. We performed MPO activity tests and immunofluorescence staining of tissue sections 24 h after modeling to determine the effects of PSP/Reg administration on neutrophil tissue infiltration. Then we found that MPO levels in the lungs (1.26 ± 0.06 U/g vs. 2.53 ± 0.07 U/g) and kidneys (0.88 ± 0.02 U/g vs. 1.8 ± 0.16 U/g) increased by over two-fold in PSP-40 mice compared with those in PBS mice, and a similar trend was also observed in the heart (0.81 ± 0.05 U/g vs. 1.22 ± 0.07 U/g) and liver (1.21 ± 0.09 U/g vs. 1.84 ± 0.42 U/g) (Figures 4A–D). Similarly, compared with PSP-40 mice, PSP-400 mice showed increased MPO levels in all four organs (*p* < 0.01). After immunofluorescence staining, neutrophil tissue infiltration was observed in the lungs, kidneys, hearts, and livers of PBS mice, whereas PSP/Reg administration markedly increased PMN tissue infiltration, which was more pronounced in mice injected with a higher dose of PSP/Reg (Figures 4E–H). These findings suggested that PSP/Reg administration enhanced neutrophil infiltration in important organs.

### 3.5. PSP/Reg promoted PMN activation induced By CLP *in vivo* and *in vitro*

**Figure 5:**
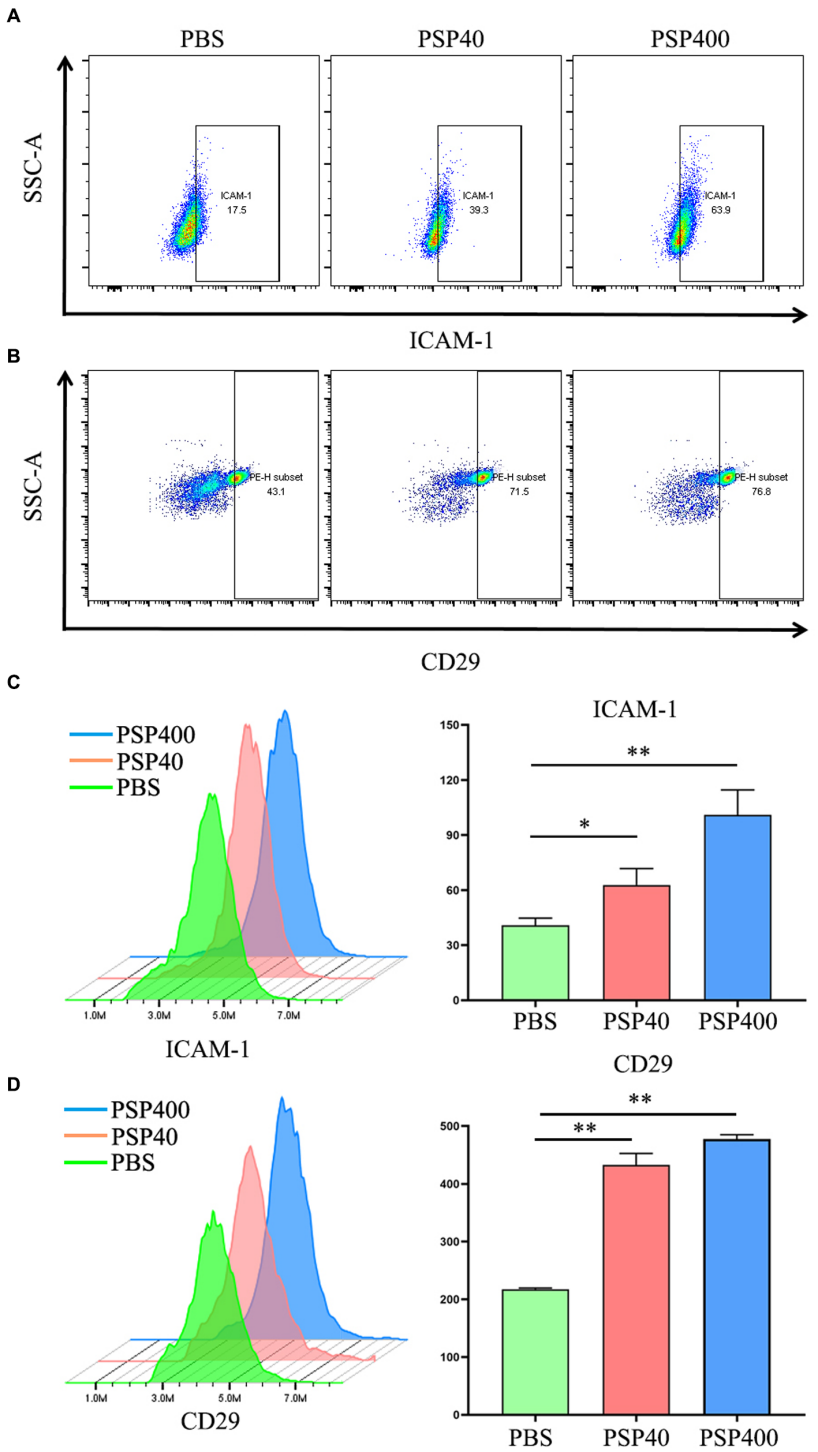
Pancreatic stone protein/regenerating protein (PSP/Reg) increased the levels of neutrophil surface adhesion molecules. Cecal ligation and puncture mice were stimulated with PSP/Reg 30 min after surgery, and 24 h later, flow cytometry was performed to detect the percentage of intercellular adhesion molecule 1 (ICAM-1)-positive **(A)** and CD29-positive **(B)** cells in mouse bone marrow neutrophils. Neutrophils were isolated from the mouse bone marrow and stimulated with 40 ng/ml and 400 ng/ml of PSP/Reg overnight. **(C)** Representative histograms and MFI quantification analysis of ICAM-1 levels in PMN under 40 ng/ml stimulation. **(D)** Representative histograms and MFI quantification analysis of CD29 levels in PMN under 400 ng/ml stimulation.The data were analyzed by one-way analysis of variance. ^*^p < 0.01, ^**^p < 0.001 compared with the PBS group.

The effect of PSP/Reg administration on the PMN status of experimental mice was analyzed by isolating neutrophils from the bone marrow of CLP-treated mice. FCM showed that PSP/Reg-injected mice showed significantly increased levels of ICAM-1 and CD29 (integrin 1β) (Figures 5A,B). To further validate the activating effect of PSP/Reg on neutrophils, they were isolated from the bone marrow of wild-type mice, cultured *in vitro*, and injected with equivalent amounts of PSP/Reg (40 or 400 ng/mL) or PBS. PSP/Reg stimulation resulted in increased levels of ICAM-1 and CD29 (Figures 5C,D), consistent with the *in vivo* findings. These results indicated that PSP/Reg administration put neutrophils in a proinflammatory state, exacerbated sepsis-induced high PMN tissue infiltration, and led to aggravated multiorgan damage.

## 4. Discussion

MODS is a devastating complication in critically ill patients that frequently leads to increased morbidity and mortality in the ICU. Despite the severe effect of MODS on patient outcomes, factors that drive its development into sepsis remain poorly understood. Further in-depth investigations are warranted to gain a better understanding of the mechanisms underlying this condition. Therefore, we designed a prospective clinical study to explore the potential correlation between PSP/Reg and the development of sepsis-induced multiple organ dysfunction. In this study, we established a mouse model of CLP-induced sepsis to investigate the possible involvement of PSP/Reg in the progression of sepsis-induced multiple organ dysfunction via neutrophil activation.

Initially, PSP/Reg was named PSP because it was found to inhibit the precipitation of calcium carbonate crystals in the pancreatic juice within the pancreatic stones of patients with chronic calcific pancreatitis (9). Subsequently, Reg promoted pancreatic tissue repair and regeneration. Because Reg and PSP were later proved to be identical, both of which are encoded by a transcript of the same REG gene and are structurally similar to the C-type lectin-like protein, the protein was renamed PSP/Reg (10). Further research on PSP/Reg has revealed its increased levels in various infectious diseases, including severe infections (11), appendicitis (12), peritonitis (13), pneumonia (14), and sepsis (15). It has also been identified as a potential biomarker for infection and sepsis. Keel et al. demonstrated that (16) PSP/Reg levels increased in 83 patients with severe trauma, but no pancreatic injuries, thus serving as a favorable predictor of infection and sepsis. Moreover, it was confirmed that the onset of sepsis could be predicted earlier via increased PSP/Reg levels than via conventional inflammatory factors, such as C-reactive protein and procalcitonin (15). Similar to previous research, the present study revealed that PSP/Reg levels, which are associated with the severity of sepsis among adult patients, could be utilized to predict patient prognosis and progression to MODS. Notably, PSP/Reg has been identified as a stress protein in both animal models and clinical experiments, indicating its potential as a target for therapeutic interventions against sepsis and related conditions (16, 17). Reding et al. revealed that (18) PSP/Reg, which can respond to injuries in early-stage infections, might be an indicator of systemic stress and a type of secretory stress protein secreted by pancreatic acinar cells after pancreatic perception of distal organ injury and systemic stress. Compelling evidence was provided to confirm that PSP/Reg levels increased during early-stage infections, burns, and traumatic diseases and were visibly associated with the severity of injuries.

In sepsis, it is difficult for neutrophils to be recruited to the site of infection (19), which makes it difficult for invading pathogens to be cleared away from the body in a timely and effective manner, leading to an overactive inflammatory response in the body. In addition, owing to the excessive infiltration of neutrophils in important distal organs, activated PMNs in the organs generate and release proinflammatory cytokines, reactive oxygen species, lysosomal enzymes, neutrophil extracellular traps, and other active substances that trigger organ injury and MODS (20). Therefore, a better understanding of the mechanisms underlying sepsis-induced organ damage caused by PMN activation and recruitment is of great importance for the development of potential therapeutic strategies. In this study, PSP/Reg injection increased the levels of systemic inflammatory factors in mice receiving CLP, exacerbated the apoptosis in their vital organs, and increased levels of organ-damage markers. In the meantime, the infiltration of PMNs in their organs was also found to be enhanced. Although the effect of PSP/Reg has not yet been clarified, its structural homology has been characterized; PSP/Reg is a type of globular polypeptide that adopts the overall folds of C-type lectins (21). C-type lectin-like proteins, calcium-dependent glycan-binding proteins, are known to function as adhesion and signaling receptors in homeostasis and innate immunity and play a crucial role in inflammatory responses as well as leukocyte and platelet trafficking (22). The protein structure of PSP/Reg also supports its interaction with neutrophils. In addition, following the incubation of PSP/Reg with human whole blood, the levels of β-2-integrins (CD11b) in blood PMNs increased, whereas those of L-selectins (CD62L) decreased; PSP/Reg could also directly bind to PMNs after incubation with purified PMNs (16). Therefore, we hypothesized that sharply increased levels of PSP/Reg during sepsis might be involved in the host response to pathogens by activating PMNs, thus playing a role in the progression to MODS.

To further verify the regulatory effect of PSP/Reg on PMN tissue infiltration, we detected the surface activation indicators of bone-marrow PMNs in mice administered with PSP/Reg and discovered that PSP/Reg stimulation increased the levels of the adhesion molecules ICAM-1 and CD29 on the surface of PMNs. The activation and tissue infiltration of neutrophils are strictly regulated by adhesion molecules (23). During inflammation, the increased levels of adhesion molecules mediate the recruitment and activation of PMNs and induce tissue infiltration through the binding of their cytoplasmic domains to actin cytoskeletons (24). Furthermore, it has been reported that the increased level of ICAM-1 in neutrophils in both human and animal models of sepsis induces membrane surface rigidity and impairs cell deformability (25), leading to their decreased ability to migrate to infectious sites, resulting in microvascular and organ dysfunction. Notably, treatment with ICAM-1-specific antibodies can reduce neutrophil migration to distant organs, such as the lungs and spleen (26). The increased level of CD29, also known as integrin beta-1, on the surface of PMNs is associated with the activation and recruitment of neutrophils during inflammation (23). The present study suggested that the activation and induction of neutrophils and inhibition of anti-inflammatory factors by PSP/Reg were partially responsible for the inflammatory responses during early-stage sepsis and multiple organ dysfunction, which also explained the relationship between PSP/Reg levels and progression to MODS in patients with sepsis.

Among the 141 patients with sepsis admitted to the ICU in our study, those with septic shock exhibited significantly higher levels of circulating PSP/Reg. There was a strong correlation between circulating PSP/Reg levels and 7-day survivors requiring organ support, indicating that disease severity was an independent risk factor for progression to MODS 48–72 h after admission and 28-day mortality. Moreover, this study showed that the intravenous injection of PSP/Reg aggravated multiorgan damage in the CLP mouse models and shortened their survival time. At the cellular level, we confirmed that PSP/Reg induced the inflammatory activation of murine neutrophils, further supporting its role in promoting inflammation during sepsis. This indicated that PSP/Reg might facilitate the activation and infiltration of neutrophils in distal organs to intensify the CLP-induced inflammatory cascade, thereby exacerbating multiorgan damage in sepsis.

Sepsis and MODS are multifaceted conditions with highly intricate pathogeneses involving a dynamic and complex immune response that may involve both proinflammatory and anti-inflammatory mechanisms. The effect of PMNs on multiorgan damage in early and late sepsis is not yet fully understood, and further studies are needed to investigate the underlying mechanisms that regulate the immune response during these phases of sepsis. Our study has shed new light on the potential role of PSP/Reg in exacerbating sepsis-induced multiple organ dysfunction by facilitating the activation and infiltration of neutrophils; the underlying regulatory mechanisms involved in this process are likely to be highly complex and multifactorial. Thus, it is necessary to explore these mechanisms in greater detail to identify potential therapeutic targets for the prevention and treatment of sepsis and MODS.

List of abbreviations: PSP/Reg, Pancreatic stone protein/regenerating protein; MODS, multiple organ dysfunction syndrome; ICU, intensive care unit; CLP, cecal ligation and puncture; PBS, phosphate-buffered saline; TUNEL, terminal deoxynucleotidyl transferase dUTP nick end labeling; MPO, myeloperoxidase; SOFA, sequential organ failure assessment; ICAM-1, intercellular adhesion molecule 1; FCM, flow cytometry; IL, interleukin; IQR, interquartile range; ROC curve, receiver operating characteristic curve; CI, confidence interval.

## Data availability statement

The original contributions presented in the study are included in the article/Supplementary material, further inquiries can be directed to the corresponding author.

## Ethics statement

The studies involving human participants were reviewed and approved by the Ethics Committee of the First Affiliated Hospital of Nanchang University (approval number: NC-2022-1-004), and each patient had signed the informed consent form prior to participation. The patients/participants provided their written informed consent to participate in this study. The animal study was reviewed and approved by Experimental Animal Welfare and Ethics Committee of the First Affiliated Hospital of Nanchang University. Written informed consent was obtained from the individual(s) for the publication of any potentially identifiable images or data included in this article.

## Author contributions

PH contributed to the experimentation, analysis, and manuscript preparation. FL contributed to the research concept and oversaw the study. QS and WD contributed to the performance of experiments. LW and WXZ contributed to the immunological evaluation through sample measurements. KQ contributed to the revision of the manuscript. YL contributed to the article by making important revisions and additions. QL contributed to the article by providing assistance with further subgroup analysis of the data. NZ contributed to the article by helping to guide the statistical analysis.

## Conflict of interest

The authors declare that the research was conducted in the absence of any commercial or financial relationships that could be construed as a potential conflict of interest.

## Publisher’s note

All claims expressed in this article are solely those of the authors and do not necessarily represent those of their affiliated organizations, or those of the publisher, the editors and the reviewers. Any product that may be evaluated in this article, or claim that may be made by its manufacturer, is not guaranteed or endorsed by the publisher.
