# Supplementary figures and images for: The critical role of pancreatic stone protein/regenerating protein in sepsis-related multiorgan failure
Source: Front Med (Lausanne). 2023 May 5;10:1172529. doi: 10.3389/fmed.2023.1172529 (PMC10196489; doi:10.3389/fmed.2023.1172529)

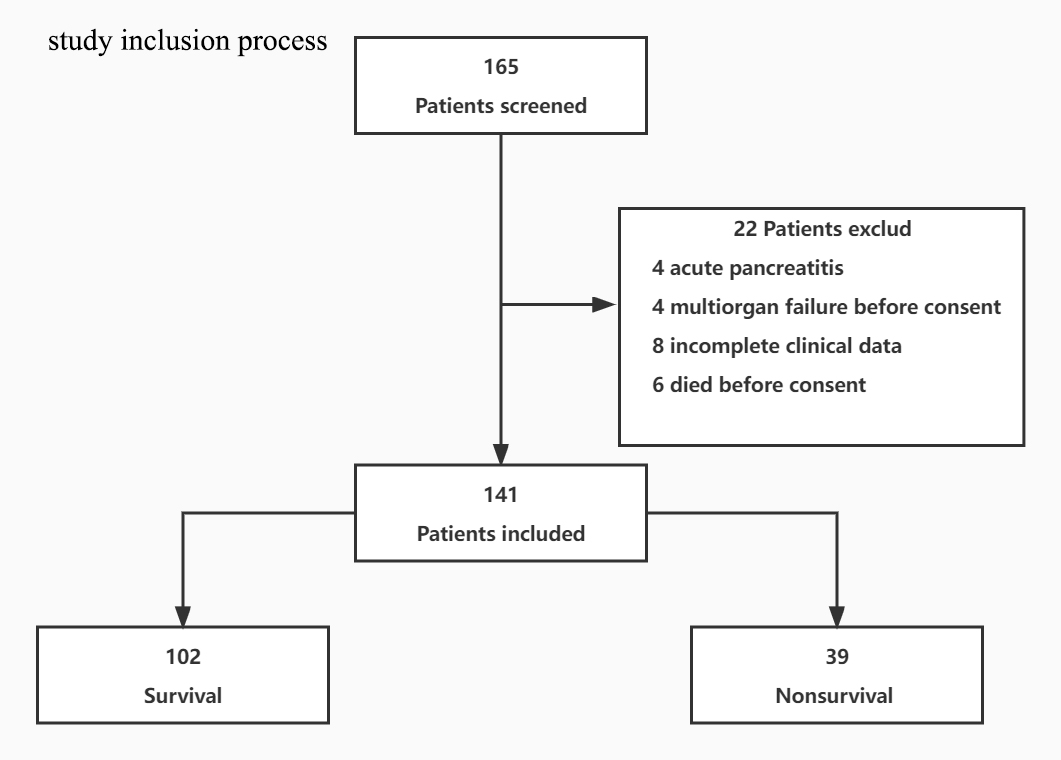

Supplement: Supplementary file 1 [file Image_1.JPEG]
