# Supplementary material for: The critical role of pancreatic stone protein/regenerating protein in sepsis-related multiorgan failure
Source: Front Med (Lausanne). 2023 May 5;10:1172529. doi: 10.3389/fmed.2023.1172529 (PMC10196489; doi:10.3389/fmed.2023.1172529)
Supplement: Supplementary file 2 [file Table_1.DOCX]

| Severe sepsis | AUC(95%Cl) | Accuracy | Sensitivity | Specificity |
| --- | --- | --- | --- | --- |
| MODS progression | 0.77(0.628-0.911) | 0.761 | 1 | 0.736 |
| 28d mortality | 0.607(0.437-0.778) | 0.619 | 1 | 0.407 |

Table2: The receiver operating characteristic curve analysis of PSP/Reg for the prediction of MODS in Severe sepsis patients
