# Supplementary material for: The critical role of pancreatic stone protein/regenerating protein in sepsis-related multiorgan failure
Source: Front Med (Lausanne). 2023 May 5;10:1172529. doi: 10.3389/fmed.2023.1172529 (PMC10196489; doi:10.3389/fmed.2023.1172529)
Supplement: Supplementary file 3 [file Table_2.DOCX]

| Sepsis shock | AUC(95%Cl) | Accuracy | Sensitivity | Specificity |
| --- | --- | --- | --- | --- |
| MODS progression | 0.645(0.512-0.778) | 0.464 | 0.904 | 0.346 |
| 28d mortality | 0.723(0.617-0.828) | 0.676 | 0.906 | 0.567 |

Table3: The receiver operating characteristic curve analysis of PSP/Reg for the prediction of MODS in Sepsis shock patients
